# Iron accumulation and lipid peroxidation: implication of ferroptosis in hepatocellular carcinoma

**DOI:** 10.3389/fendo.2023.1319969

**Published:** 2024-01-04

**Authors:** Xiaodong Li, Fanguang Meng, Hankang Wang, Liwei Sun, Shulin Chang, Guijie Li, Feng Chen

**Affiliations:** ^1^ Department of Radiology, The First Affiliated Hospital of Shandong First Medical University and Shandong Provincial Qianfoshan Hospital, Shandong Medicine and Health Key Laboratory of Abdominal Medicine Imaging, Jinan, China; ^2^ Graduate School, Shandong First Medical University and Shandong Academy of Medical Sciences, Jinan, China

**Keywords:** ferroptosis, hepatocellular carcinoma, chemotherapy, drug resistance, therapy

## Abstract

Ferroptosis is a type of controlled cell death caused by lipid peroxidation, which results in the rupture of the cell membrane. ferroptosis has been repeatedly demonstrated over the past ten years to be a significant factor in a number of diseases. The liver is a significant iron storage organ, thus ferroptosis will have great potential in the treatment of liver diseases. Ferroptosis is particularly prevalent in HCC. In the opening section of this article, we give a general summary of the pertinent molecular mechanisms, signaling pathways, and associated characteristics of ferroptosis. The primary regulating mechanisms during ferroptosis are then briefly discussed, and we conclude by summarizing the development of a number of novel therapeutic strategies used to treat HCC in recent years. Ferroptosis is a crucial strategy for the treatment of HCC and offers new perspectives on the treatment of liver cancer.

## 1 Introduction

Hepatocellular carcinoma (HCC) is the most frequent malignant tumor of the liver, with the highest occurrence among people with chronic liver disease (1) and a dismal 5-year survival rate. Although the number of therapeutic options for HCC has gradually increased in recent decades, including surgical resection, immunotherapy, targeted therapy, or combination therapy, overall survival and prognosis remain limited due to the lack of specific symptoms in the early stages of the disease and the loss of therapeutic opportunities (2). As a result, it is critical to investigate the pathophysiology of hepatocellular carcinoma as well as the regulation of signaling pathways in order to uncover new therapeutic targets and develop safe and effective therapeutic choices.

Ferroptosis is an iron-dependent controlled cell death caused by polyunsaturated fatty acid-containing phospholipids (PL-PUFA) in cell membranes that differentiates from other types of cell death by iron-dependent accumulation of lipid peroxides and redox imbalance (3). Numerous investigations have found that numerous diseases, including neurological diseases, autoimmune diseases, liver diseases, and malignancies, are intimately associated to ferroptosis (4). During carcinogenesis, ferroptosis prevents the formation and proliferation of HCC cells. However, HCC cells create specialized defensive mechanisms to prevent ferroptosis and hence continue tumor growth. The cell death mode process is changed by targeting ferroptosis-related regulatory components. We outline the ferroptosis-related pathways and important influencing factors in this research in order to give new therapeutic methods for patients.

## 2 The mechanism of ferroptosis

**Figure 1:**
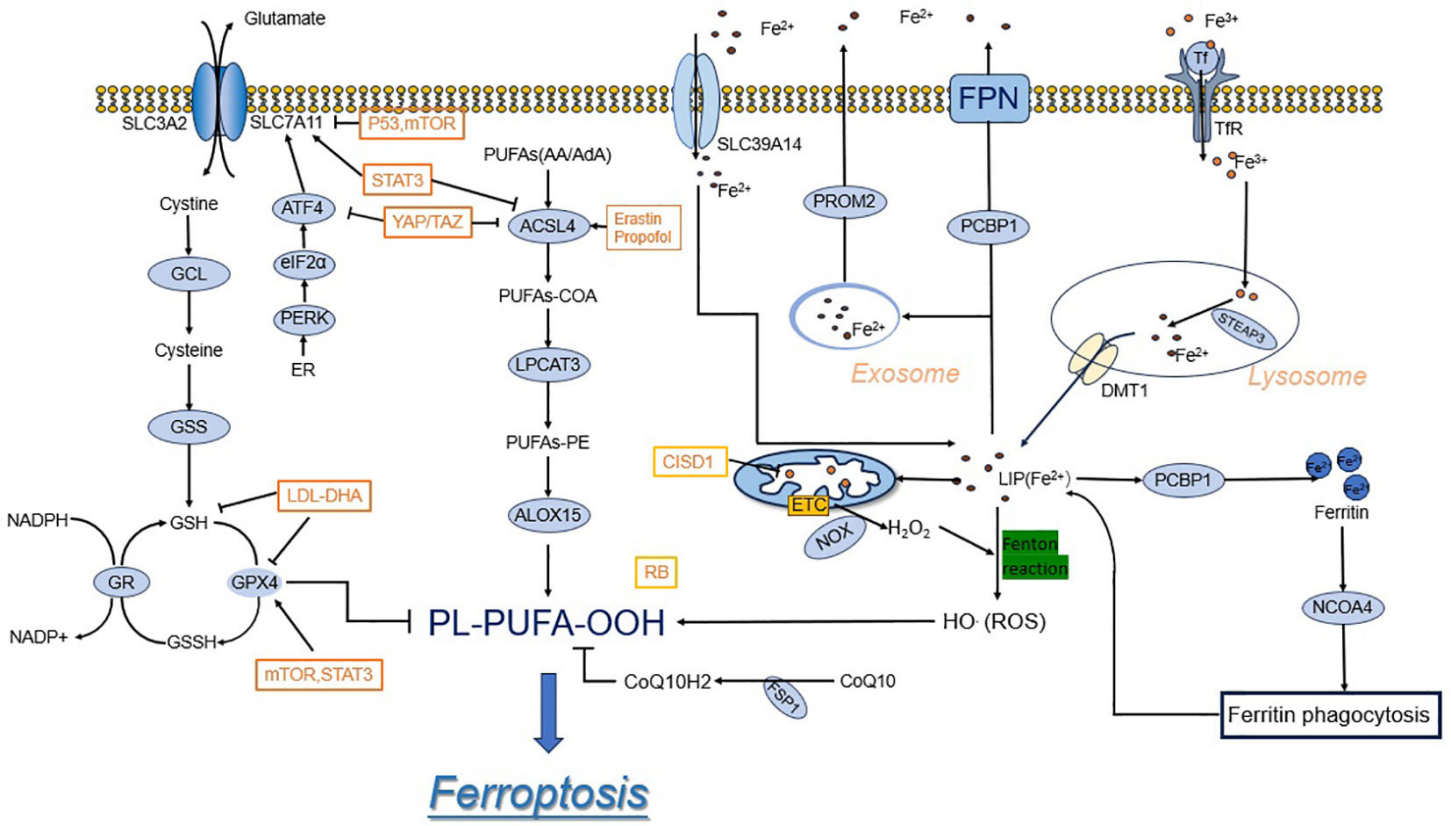
Ferroptosis mechanisms and regulations. System xc- imports cystine, which is reduced in the cell to the amino acid cysteine. Cysteine and glutamate are used in the biosynthesis of reduced glutathione, which is in turn used by GPX4 to reduce reactive PUFA phospholipid hydroperoxides (PUFA-PL-OOH) to non-reactive and non-lethal PUFA phospholipid alcohols (PUFA-PL-OH). Iron is present in LIP in the form of Fe^2+^, which promotes Ferroptosis. In the top panel, acetyl-CoA is used to make free PUFAs, which are activated by ACSL4, LPCAT3, and ACSL1 to generate PUFA-PLs.

The rise in ROS surpasses the regulating capacity of glutathione (GSH) and GPX 4, resulting in lipid peroxidation (5). The morphological aspects of ferroptosis are characterized by changes in mitochondrial morphology and mitochondrial cristae structure, such as mitochondrial atrophy, increased membrane density, decreased mitochondrial cristae, and outer mitochondrial membrane rupture. In addition, unlike other cell deaths, ferroptosis cells do not demonstrate reduced cell size, nucleus abnormalities, chromatin aggregation, or cytoskeletal disintegration (3, 6–8). At the cellular level, ferroptosis is triggered by the inhibition of cell membrane translocation factors such as cystine/glutamate translocator (also known as the system Xc-) or the activation of transferrins, as well as the blockade of intracellular antioxidant enzymes such as glutathione peroxidase 4 (GPX4) (3, 9). In this article, a synopsis discusses the physiology of ferroptosis and summarizes the relevant targets and mechanisms of action (**Figure 1**).

### 2.1 Reactive oxygen species metabolism

The peroxidation of lipids is an advantageous feedback chain reaction initiated by free radicals; these ROS, including superoxide (O2), peroxide (H2O2 and ROOH), and free radicals (HO and RO), catalyze polyunsaturated fatty acid oxidation (10). Mitochondria are the main producer of intracellular ROS, i.e., partial reduction inside the respiratory chain of the mitochondria (11, 12). Additionally, ROS are produced by enzymes in the endoplasmatic reticulum, peroxisomes, and the plasma membrane (such as NOX and NADPH oxidases), and additionally non-enzymatically via the Fenton reaction (13, 14). Under the catalyst effect of Fe^2+^, hydrogen peroxide creates hydroxyl radicals (HO), which is the foundation of free radical lipid peroxidation, known as the Fenton reaction. The two most common ROS that impact lipids are hydroxyl radicals (HO) and hydroperoxides (ROO) (15).

The GSH/GPX 4 axis prevents iron-catalyzed apoptosis, whereas cysteine (cys), an inhibitory amino acid in GSH synthesis that blocks cys input via SLC7A11, causes ferroptosis by lowering GSH levels.

#### 2.1.1 System Xc^-^

Solute carrier family 7 member 11 (SLC7A11, catalytic subunit) and solute carrier family 3 member 2 (SLC3A2, regulatory subunit) make up System Xc- (3). The cell membrane’s System Xc- maintains redox equilibrium by absorbing external cys and excreting it in a proportion equal to one to intracellular glu (16). Cysteine is readily transformed to cysteine after entering the cell via System Xc-. Cysteine is an inhibitory amino acid that inhibits GSH production. GSH is a widespread endogenous antioxidant that exists in both reduced (GSH) and oxidized (GSSG) forms, and the GSH/GSSG ratio shows the level of cellular oxidative stress. Ferroptosis was accelerated by lowering intracellular cysteine levels and hence GSH concentration, which increased lipid peroxidation. Furthermore, SLC7A11 enhances cystine absorption and glutathione production, which protects cells from oxidative stress and ferroptosis. SLC7A11 is regulated by two transcription factors, nuclear factor erythroid 2-related factor 2 (NRF2) and activator of transcription 4 (ATF4), according to extensive research.

The antioxidant response is mediated by NRF 2, a master transcription factor. NRF 2 interacts with kelch-like ECH-associated protein-1 (KEAP1) and targets KEAP1-Cullin3-mediated polyubiquitination and proteasomal degradation under non-stress settings (17). Oxidative stress inducers, such as oxidizing agents and electrophilic chemicals, cause KEAP1 oxidation, which inhibits NRF2 degradation by the KEAP1-Cullin3 ubiquitin ligase complex. The stabilized NRF2 then translocates into the nucleus, attaches to antioxidant response elements (AREs) in gene promoters, and regulates the transcription of target genes involved in antioxidant responses and cellular redox maintenance. Several antioxidant response elements (AREs) were discovered after analyzing the SLC7A11 promoter. Further investigation revealed that pro-electronic reagents and other cellular stressors stimulate SLC 7A 11 expression by tethering NRF 2 to antioxidant response elements (AREs) in the SLC 7A 11 promoter (18). As consequence, NRF2 overexpression increases the expression of SLC7A11 as well as other antioxidant target genes, resulting in enhanced GSH production (19). SLC7A11 is thus one of the NRF 2 transcriptional targets mediating antioxidant responses. ATF 4 is a transcription factor that controls redox homeostasis, amino acid metabolism, and ER stress (20). Unlike NRF 2, which is stabilized in response to stress, ATF 4 mRNA translation is accelerated under varied stress circumstances. Eukaryotic initiation factor 2α (eIF2α) is a regulator that comes before ATF4. eIF2α phosphorylation stimulates ATF4 mRNA translation and increases ATF4 protein levels (20). General control non-derepressible-2 (GCN2) is an upstream kinase of eIF2α that is triggered by amino acid deprivation. As a result, amino acid deficiency stimulates GCN 2, which phosphorylates and inactivates eIF2α, resulting in enhanced ATF4 protein production. ATF4 then attaches to amino acid response elements (AARE) in gene promoters, regulating transcription of genes involved in amino acid metabolism and stress response (21). Specifically, ATF4 binds to the AARE in the SLC7A11 promoter, causing SLC7A11 expression (22).

Normally, ATF4 and NRF2 work together to control gene expression. For instance, under stressful circumstances, ATF4 and NRF2 interact at the SLC7A11 promoter and work together to synergistically control SLC7A11 transcription (23). Additionally, under certain situations that cause iron-catalyzed apoptosis, p53 can accelerate iron apoptosis by suppressing SLC7A11 expression, whereas p53 deficiency-induced increases in SLC7A11 decrease ferroptosis (24). ATF3, another transcription factor from the ATF/CREB family, has been found to bind to the SLC7A11 promoter and restrict its expression independently of p53, and ATF 3 accelerates erastin-induced ferroptosis by down-regulating SLC7A11 levels (25). ATF3 primarily regulates SLC7A11 transcription under basal conditions and has no effect on stress-induced SLC7A11 expression. In summary, various stress circumstances enhance SLC7A11 transcription in part via ATF4 and/or NRF2, whereas p53 and ATF3 mostly suppress SLC7A11 expression under normal conditions. Thus, these transcription factors change cellular vulnerability to ferroptosis by regulating SLC7A11 expression.

#### 2.1.2 GPX4/GSH

Ursini et al. discovered GPX4 as a selenoenzyme in 1985, making it the sole member of the PL-OOH scavenger family capable of turning harmful lipid hydroperoxides into harmless phosphatidyl alcohols (26). GPX4’s primary role as a major antioxidant peroxidase is to use GSH as a cofactor to resist lipid peroxidation and hence safeguard membrane integrity. GPX4 interacts with the polar head of phospholipids, allowing it to bind to bilayer membranes, according to structural and biochemical investigations (27). GSH is utilized as a cofactor by GPX4 to convert membrane lipid peroxides into harmless lipid alcohols (28). GPX4 uses two GSH molecules as donors to reduce a phospholipid hydroperoxide (PL-OOH) molecule to an alcohol molecule, producing GSSG, which can be converted to GSH utilizing NADPH/H+ and glutathione reductase (29). Overall, the resistance to ferroptosis of GPX4 requires catalytic selenocysteine residues and depletion of GSH, and inhibition of peroxide accumulation is achieved by GSR/GSH (glutathione disulfide reductase) and NADPH/H+ recirculation. GPX4 protects cells from oxidative damage by lowering thymine hydroperoxides, cholesterol hydroperoxides, and fatty acid hydroperoxides in addition to utilizing GSH to remove harmful PL-OOH (30, 31). Reactive oxygen species (ROS) build up in cells and trigger ferroptosis when GPX4 is suppressed (32). GPX4 might perform a special protective role in growth and development. In a mouse model, Yant et al. showed that gpx 4-/- embryos died *in utero* at mid-gestation (33, 34). The antioxidant function of GSH, which is mostly made up of glutamate, cysteine, and glycine, is primarily found in mammalian cells. These amino acids have sulfhydryl structures that can be oxidized, which enables GSH to shield cells from oxidative stress damage. The selenoenzyme GPX4 functions as a cofactor for GSH, which causes ferroptosis by inhibiting GPX4 synthesis from scratch (35). While altering the enzymatic activity of GPX4, glutamine-cysteine synthase activity, cysteine concentration, and GSH feedback inhibition directly control GSH production. On the one hand, GPX4 is transcriptionally regulated. By boosting GSH cell levels, nuclear factor erythroid-2-related factor 2 (NRF2) enhances GPX4 activity (36). To maintain redox equilibrium and promote carcinogenesis, oncogenic KRAS stimulates the Nrf2 antioxidant system (37). Through metabolic reprogramming, NRF2 activation imparts chemoresistance in KRAS-driven cancer cells (38). NRF2 inhibits lipid peroxidation and ferroptosis by activating antioxidant-responsive element (ARE)-containing genes involved in GSH production, glutamine metabolism, and iron metabolism (36). GSL2 is a mitochondrial aminotransferase and a direct transcriptional target of P53, which binds to the corresponding element in the GLS2 gene and induces transcription of GLS2, which inhibits ferroptosis by increasing cellular antioxidant capacity by promoting the production of GSH and NADPH (39, 40), but has no direct effect on GPX4 expression through binding the GPX4 promoter (41). Meanwhile, as previously described, cystine uptake was reduced after SLC7A11 was inhibited as a direct target of P53, which in turn affected GSH biosynthesis (24).

On the other hand, different chemicals influence GPX4 synthesis at the post-transcriptional stage. In general, GPX4 homeostasis is affected by both selenocysteine biosynthesis/uptake (35, 42) and GSH synthesis (43, 44),implying that GPX4 is important in regulating ferroptosis. SLC7A11 overexpression is detected in many human malignancies, and inhibiting the SLC7A11-GSH axis has been shown to have an anticancer effect (45). As a result, the cyst(e) ine/GSH/GPX4 axis is crucial for avoiding lipid peroxidation and ferroptosis. Cancerous tumors require significant levels of cysteine and GSH to counteract the increased intracellular ROS and nutritional dependency required for the enhanced action of SLC7A11 (46). A lack of intracellular cysteine, GSH, or SLC7A11 suppression reduces GPX4 activity, which can enhance lipid peroxidation and trigger ferroptosis (47).

### 2.2 Lipid peroxidation

Lipid peroxidation is a biomarker of ferroptosis (48). By changing the lipid composition of cellular membranes, abnormal lipid metabolism increases lipid peroxidation and promotes ferroptosis (49). During ferroptosis, acyl-CoA synthetase long-chain family member 4 (ACSL 4), lysophosphatidylcholine acyltransferase 3 (LPCAT 3), and arachidonic acid 15-lipoxygenase (ALOX 15) are required for lipid peroxidation. Arachidonic acid (AA), epinephrine (AdA), and phosphatidylethanolamines (PE), which are necessary for ferroptosis, have been found by quantitative lipidomics (50). ACSL4 encodes for the formation of proteins that link acetyl coenzyme A to AA during lipid synthesis to generate long-chain polyunsaturated lipoyl coenzyme A (AA-CoA). In the presence of LPCAT3, AA-CoA then interacts with PE to generate PE-AA, which then enters the lipid oxidation pathway. In the presence of LOX, PE-AA produces phospholipid peroxides. The process described above happens when ACSL4 and LPCAT3 dope PUFA (for example, arachidonic acid, AA) into membrane-localized lipids, which must be present in a membrane-bound milieu for PUFA to be lethal following peroxidation (51). Phospholipid peroxide accumulation depletes intracellular COQ10 and GSH on the one hand while increasing cellular vulnerability to ferroptosis on the other. Thus, it is clear that lipid anabolism works as a precursor to and regulator of lipid oxidation metabolism (52).

#### 2.2.1 ACSL 4/LPCAT 3

A synthetase long-chain family (ACSL) is a major enzyme involved in lipid metabolism *in vivo*, notably facilitating the production of fatty acids with carbon chains ranging from 12 to 20 (53). In mammalian cells, there are five isozymes (ACSL 1, ACSL 3, ACSL4, ACSL5, ACSL6), and different isozymes play different roles in different tissues. ACSL4 is the key enzyme catalyzing the activation of long-chain fatty acids in ferroptosis, and its aberrant expression is closely associated with a number of biological responses, including steroidogenesis, inflammatory responses, cell death, and immune activation responses (54). The endoplasmic reticulum (ER) and the outer mitochondrial membrane (OMM) are the primary locations of the ACSL4 protein. In particularly, ACSL4 plays a crucial role in regulating ferroptosis, and its overexpression boosts the synthesis of -pufa6 PUFA and encourages ferroptosis (51).

Meanwhile, ACSL4 is regulated by multiple factors, such as integrin α6β4-mediated inhibition of ACSL 4 expression by Src and signal transducer and activator of transcription 3 (STAT3) or activation of the androgen receptor (55). It’s noteworthy to observe that the presence of free AA can alter the levels of ACSL4 by encouraging its ubiquitination and proteasomal destruction (56). The transcription of ACSL4 is started by a cAMP-response element binding (CREB) site found in the proximal promoter region of the gene (57). By encouraging ferroptosis by releasing YAP’s activity, the ACSL4 gene can begin to transcribe (58). In HCC cells, where ACSL4 is overexpressed and is associated with lower overall survival and disease-free survival (DFS) in patients with the disease, ACSL4 also plays a significant role. Aspirin and sorafenib together, as shown by Xia et al., synergistically triggered apoptosis in HCC cells by inhibiting ACSL4 expression (59).

LPCAT3 is a member of the LPLAT family of lysophospholipid acyltransferases. Among them, LPCAT3 dopes PUFA-CoA into phospholipids primarily in the endoplasmic reticulum (ER). A haploid gene screen for LPCAT3 found it in cells exposed to non-apoptotic cell death-inducing small compounds (60). The discovery of genes involved in lipid metabolism, such as ACSL4, during this screening lends credence to the notion that PUFA metabolism is fundamental to ferroptosis. In mouse lung epithelial (MLE) cells and mouse embryonic cells, Lpcat3 knockdown results in resistance to RSL3-induced ferroptosis, which may demonstrate the crucial function of LPCAT3 in ferroptosis (50, 60).

#### 2.2.2 ALOX

Lipid peroxidation, which is mediated by either cytochrome P450 oxidoreductase (POR) (61) or lipoxygenase (ALOX) (62, 63), is the last stage in the generation of lipid peroxides. Humans have six ALOX genes, including ALOX5, ALOX12, ALOX12B, ALOX15, ALOX15B, and ALOXE3, which influence ferroptosis in different ways depending on the situation (64). ALOX12, for example, promotes TP53-dependent iron apoptosis in specific cancer cells (61). In bronchial epithelial cells, renal epithelial cells, and neurons, ALOX15 binds to phosphatidylethanolamine-binding protein 1 (PEBP1) and triggers RSL3-induced ferroptosis (65). ALOX5 is involved in ferroptosis triggered by erastin or RSL3 (66), which can be suppressed by its binding protein microsomal glutathione S-transferase I (MGSTl) (67).

### 2.3 Iron metabolism

**Figure 2:**
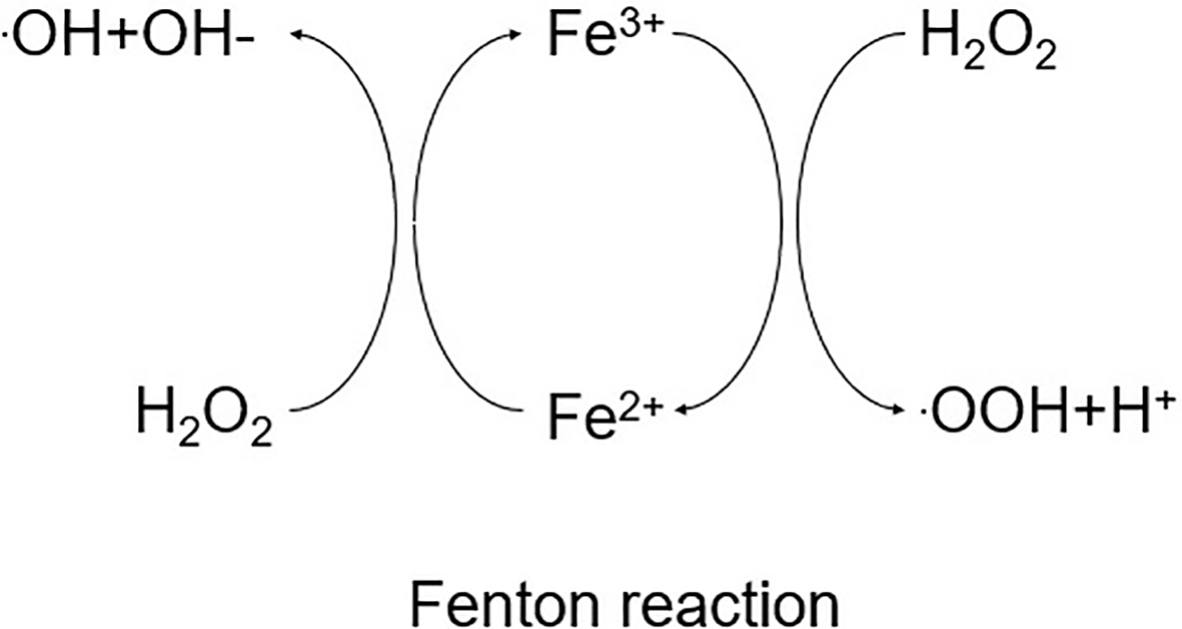
Schematic diagram of the Fenton reaction.


Iron is a trace element that exists in two forms in the human body: divalent iron (Fe^2+^, absorbable) and trivalent iron (Fe^3+^, non-absorbable) (68, 69). The liver is the primary organ in charge of iron metabolism, delivering and regulating the iron required for metabolism throughout the body, and there are two pathways for iron uptake: transferrin-bound iron (TBI) and non-transferrin-bound iron (NTBI). Under normal conditions, Fe^2+^ is largely imported by membrane iron transport protein (FPN) on the basolateral membrane of the duodenum, where it is oxidized to Fe^3+^ and then collected by transferrin (Tf) in plasma (70). Transferrin is a plasma glycoprotein generated by hepatocytes and transported to organs throughout the body, where it chelates circulating Fe^3+^ safely (70). The iron-loaded transferrin is then recognized by the cell membrane receptor TfR1 and translocated into endosomes via the receptor-mediated endocytosis pathway, where ferric ions are released from the transferrin in acidic endosomes and then reduced to Fe^2+^ by the enzyme prostate 3 metal reductase (STEAP 3) (71). But in conditions of iron overload, plasma transferrin’s iron-binding capability is exceeded, resulting in NTBI. Through the presence of divalent metal transporter protein 1 (DMT1, also known as soluble protein carrier [SLC11A2]) and ZRT,IRT-related protein 14 (ZIP14, also known as SLC39A14), Fe^2+^ can be transferred into the cytoplasm as an unstable iron pool (LIP) (72). To balance the redox state and intracellular iron homeostasis, excess intracellular Fe^2+^ can be translocated out of the cell via the carrier SLC40A1 (73, 74). The remainder of the iron leaves the cell via membrane iron transport protein (FPN). Ferritin is a cytosolic protein with two subunits (FTL and FTH1) that serves as the primary protein for iron storage (as Fe^3+^) (75). Iron metabolism can regulate ferroptosis via a variety of methods. Excess iron is retained in ferritin during iron overload, where it binds nuclear receptor coactivator 4 (NOCA4). NOCA4 attaches to ferritin and is destroyed by ferritin phagocytosis (76), eventually releasing free iron to participate in the Fenton reaction, which generates ROS and increases the accumulation of intracellular lipid peroxides (LPOs) and ferroptosis (77) (**Figure 2**).

$$\text{Fenton reactio}:{\text{Fe}}^{2+}+\text{HOOH}/{\text{Fe}}^{3+}+{\text{OH}}^{-}+\text{OH}$$

Several studies in recent years have shown that silencing TRFC (the gene encoding TFR1) inhibits erastin-induced iron apoptosis and prevents LIP accumulation, whereas the heme oxygenase HO-1 increases intracellular free iron levels and promotes heme degradation, which speeds up the ferroptosis process (78, 79). Furthermore, inhibiting iron response element binding protein 2 (IREB2) using shRNA changes the expression of various iron genes (e.g., TRFC, FTH1, and FTL), affecting iron absorption, metabolism, and storage (3). LIP is coordinated in the cytoplasm by a complex buffer of both small and large molecules, the main proteins of which are poly-r(C)-binding protein 1 (PCBP1) and PCBP2. They are required for iron chaperone action and are in charge of iron transport to ferritin. PCBP2 has also been demonstrated to preferentially interact with the iron transporter proteins divalent metallotransporter1 (DMT1) and membrane iron transporter protein to increase their iron import and export activities (80, 81). Sun et al. (78) previously established that heat shock protein beta-1 (HSPB1) is a negative regulator of ferroptosis in cancer cells. In cancer cells, erastin, a particular iron apoptosis-inducing chemical, increases heat shock factor 1 (HSF1)-dependent HSPB1 expression. HSF1 and HSPB1 knockdown promotes erastin-induced ferroptosis, but heat shock preconditioning and HSPB1 overexpression prevent erastin-induced ferroptosis. The activation of HSPB1 by protein kinase C decreased ferroptosis by lowering iron-mediated generation of lipid reactive oxygen species. Iron can also be exported by prominin-2 (prom 2)-mediated multivesicular bodies (MVB) of iron-containing proteins, and exosomes improve resistance to ferroptosis by depleting cells of iron and their potential to trigger lipid peroxidation (82). Under oxidative stress conditions, NRF2 separates from Keap1 and activates the expression of downstream target genes containing antioxidant response elements (ARE), such as iron heavy chain protein (FTH1) and iron light chain protein (FTL), promoting GSH synthesis and regenerating NADPH to exceed ROS production (36, 83). NRF2 stimulates the production of heme oxygenase-1 (HO-1) and the accumulation of Fe^2+^ in LIP as well as the release of iron from hemoglobin, hence inducing ferroptosis.

## 3 Mechanisms of ferroptosis regulation

There are at least three major regulatory mechanisms that play important roles in the ferroptosis process-the antioxidant regulator NRF2, the transsulfuration pathway, and mechanistic target of the rapamycin (mTOR) (84).

### 3.1 Inhibition of NRF2 triggers ferroptosis

**Figure 3:**
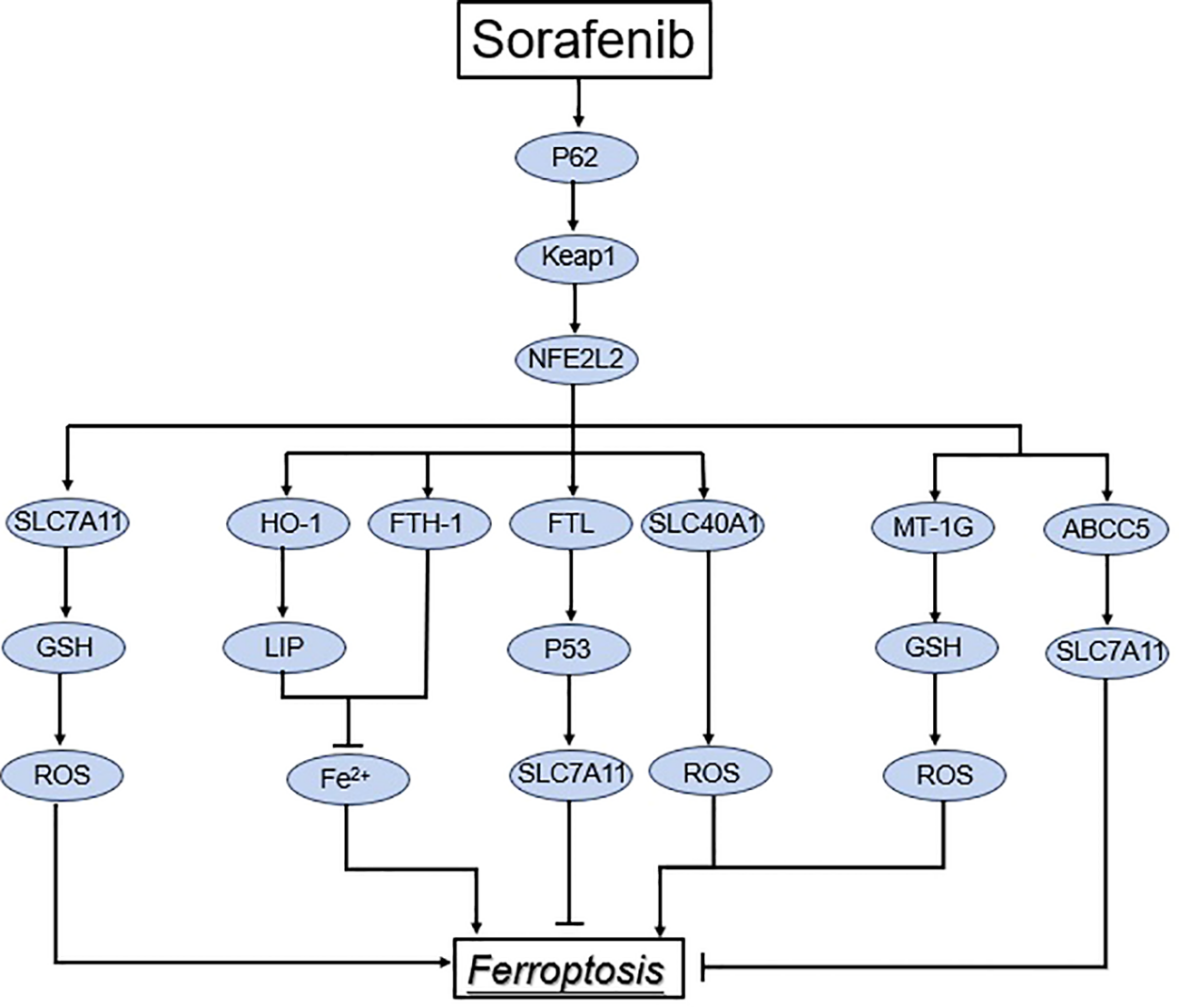
Regulation of the NRF2 pathway in ferroptosis. Under normal conditions, keap1 in association with CUL3 induce proteasomal degradation of NRF2. However, under stress conditions, keap1 cysteine residues are altered, leading to NRF2 dissociation from Keap1. As a result, NRF2 translocates to the nucleus and transactivates genes involved in the suppression of lipid peroxidation and ferroptosis.

Among the numerous mechanisms that regulate ferroptosis, NRF2 is a significant regulator of the antioxidant system, and its regulation of SLC7A11 has already been discussed. (**Figure 3**) Higher levels of oxidative stress are caused by excessive metabolic and proliferative loads in cancer cells (85). A transcription factor called NFE2-related factor 2 (NRF2) combines information from stressed cells and controls different transcriptional elements (86). NRF2, however, is no longer ubiquitinated and degraded in response to elevated oxidative stress or mutations in KEAP1, CUL3, or NRF2; this enables translated NRF2 to be translocated to the nucleus and stimulate transcription of genes with antioxidant response elements (AREs) (87, 88). NRF 2 transcriptionally induces the expression of several antioxidant defense proteins involved in iron and ROS metabolism, such as SOD1, GPX4, glutathione synthetase (GCL and GSS), HO-1, and FTH 1 (86–88). Under conditions of oxidative stress, NRF2 associates with Keap1 and activates the expression of downstream target genes containing AREs, such as ferric heavy chain proteins (FTH1) and ferric light chain proteins (FTL), which encourages GSH synthesis and regenerates NADPH to compete with ROS production (36, 83). A NRF2’s downstream antioxidant enzyme, NAD(P)H-quinone oxidoreductase 1 (NQO1), catalyzes the conversion of quinone to hydroquinone while lowering the formation of semi-hydroquinone and ROS. Meanwhile, NRF2 activated heme oxygenase-1 (HO-1) expression, which encouraged Fe^2+^ buildup in LIP and iron release from hemoglobin, as well as iron apoptosis development. A study of the drug resistance mechanism of sorafenib in hepatocellular carcinoma (HCC) cells discovered the first evidence that NFE2L2 inhibits ferroptosis (87). Sorafenib and erastin can activate the NFE2L2 pathway, resulting in the expression of antioxidant genes such as quinone oxidoreductase 1 (NQO1) and metallothionein 1G (MT1G) in HCC cells (87, 89). ABCC5, an ATP-binding cassette (ABC) protein, is a member of the ATP-dependent transporter family (90). ABCC5’s primary biological role is to expel xenobiotics and certain endogenous metabolites from cells. Sorafenib upregulates ABCC5 via the PI3K/Akt/Nrf2 pathway, which inhibits lipid peroxidation-mediated ferroptosis and promotes cancer growth, resulting in acquired sorafenib resistance in human HCC cells, according to Huang et al (91). Furthermore, ABCC5 overexpression increased SLC7A11 expression, decreased lipid peroxidation, and increased intracellular GSH levels, resulting in reduced ferroptosis (91). NRF2 activation increases the expression of the metallothionein-1 (MT1) family’s MT1G mRNA. MT1G knockdown enhances glutathione (GSH) depletion and lipid peroxidation, resulting in iron apoptosis (92). The cytosolic pro-oxidant quiescent sulfhydryl oxidase 1 (QSOX1) also enhanced ferroptosis in HCC cells by inhibiting NRF2. GSTZ1 loss in HCC can activate NRF2-associated pathways, according to recent research. GSTZ1 is thought to act as a tumor suppressor in HCC due to its reported downregulation, which results in a worse prognosis for patients and increased carcinogenesis (93, 94). Li et al. demonstrated that GSTZ1 downregulation resulted in decreased GSH and elevated ROS levels, which activated NRF2-associated pathways (94). CDGSH iron sulfur domain 2 (CISD2) is a protein that contains iron and sulfur. Sorafenib-induced ferroptosis in resistant cells was boosted by inhibiting CISD2 via ferritinophagy or the p62-Keap1-NRF2 pathway (95). Sorafenib-induced ferroptosis and cell death is aided by the downregulation of complexin II (CPLX2) and haloperidol (a sigma receptor 1 antagonist) (96, 97).

### 3.2 The transsulfuration pathway

When the System Xc-GSH-GPX4 pathway is blocked, the transsulfuration pathway acts as an alternative antioxidant process to limit lipid peroxidation. The transsulfuration (TSS) route promotes ferroptosis resistance by generating cys from methionine, which is negatively controlled by cysteyl-tRNA synthetase 1 (CARS1) (84, 98). Methionine is converted to S-adenosylmethionine (SAM) by methionine adenosyltransferase (MAT), which then creates S-adenosylhomocysteine (SAH) via TSS.SAH is converted to homocysteine (HCY), a precursor of cysteine, by S-adenosylhomocysteine hydrolase (SAHH) (99, 100). Homocysteine, a metabolite of the transsulfur pathway, has been found to promote iron apoptosis rather than promote iron resistance (101). As opposed to serving as a source of cysteine, this shows that homocysteine can act independently (84). In hepatocellular carcinoma (HCC) cells, post-translational activation of cystathionine -synthase (CBS) under TNF-induced oxidative stress increases TSS activity through a proteolytically cleaved and highly active form of CBS, increasing cystathionine and GSH production (102), which in turn promotes tumor progression and inhibits ferroptosis. Using either CBS knockdown or the cystathionase (CTH) inhibitor propargylglycine (PAG) to block the TSS pathway significantly increases ferroptosis in tumor cells (98).

While mTORC2 primarily promotes cell proliferation and survival through the phosphorylation of AKT, many tumors need mTORC1 to maintain their ability to control protein synthesis and cell growth, as well as to coordinate nutrient and energy supply to ensure that they undergo unrestricted cell division (103). Mammalian target of rapamycin complex 2 (mTORC2; also known as mechanotarget of rapamycin complex 2) is a serine/threonine kinase made up of several protein components, including mTOR, and has the ability to phosphorylate a number of downstream targets to integrate upstream growth factor stimulation with cellular processes (such as cell survival) (104). By modifying SLC7A11, the mammalian target of rapamycin (mTOR) complex family of proteins controls iron apoptosis (46). High cell density, on the other hand, inhibits mTORC1 and encourages SLC7A11 lysosomal degradation while improving SLC7A11 protein stability by lowering lysosomal degradation (46). Additionally, via phosphorylating SLC7A11 at serine 26, mTORC2 reduces the functioning of the SLC7A11 transporter (105). According to Zhang et al. (106), the mTOR pathway increases GPX4 protein synthesis and sterol response element binding protein (SREBP)-mediated lipogenesis to encourage resistance to ferroptosis (107). Hepcidin transcription regulation is linked to the pathways that react to mitogen stimulation and nutritional status through components of Ras/RAF MAPK and mTOR signaling. Additionally, mTOR is crucial for iron metabolism. In murine primary hepatocytes, mTOR and low doses of RAF inhibitors promoted ferredoxin mRNA expression (108).

### 3.3 Mechanistic target of the rapamycin

Particularly, when ACSL4 was overexpressed, mTOR phosphorylation greatly increased, and when ACSL4 was downregulated, mTOR phosphorylation significantly decreased. Rapamycin therapy also preserved the function of ACSL4 overexpression in increasing cell proliferation and preventing cell death (109). Additionally, according to Chen et al. (110), curcumin promoted iron apoptosis in colorectal cancer by controlling the expression of vital iron apoptosis indicators Fer 1, SLC7A11, GSH, MDA, and ROS through the PI3K/mTOR pathway. Recent studies have shown that the mTOR-related signaling system can either promote or inhibit ferroptosis in hepatocellular carcinoma cells (111, 112). For instance, HU et al. (113) found that miR-21-5p controlled the AKT/mTOR signaling pathway through MELK *in vivo*, reducing iron apoptosis in hepatocellular carcinoma cells. They did this by using a subcutaneous tumorigenic model in nude mice. Additionally, by phosphorylating p62, mTORC1 facilitates the binding of p62 and Keap1, which causes Keap1 to degrade and NRF2 to accumulate (107).

## 4 Ferroptosis in HCC therapy

A variety of ferroptosis inducers exist in hepatocellular carcinoma therapy. In the following narrative, we summarize the compounds that have become more popular in HCC therapy in the last few years, which are able to inhibit HCC cells in different pathways from ferroptosis, and may play a great role in the future of hepatocellular carcinoma therapy (**Table 1**).

**Table 1:** Ferroptosis inducer and their major mode of action in HCC.

Compound	Target	Mechanism	Ref
Sorafenib	SLC7A11	Inhibition of system xc-	(114)
QSOX1	NRF2	Inhibition of NRF2	(115)
Artesunate	Fe^2+^	↑LPO; ↓GSH; Ferritin degradation	(116)
Saikosaponin A	ATF3	Inhibition of SLC7A11	(117)
Erastin	SLC7A11	Inhibition of system xc-	(118)
RSL3	LIP	Inhibition of GPX4	(119)
Tiliroside	NRF2	Inhibition of NRF2	(120)
Haloperidol	S1R	Inhibition of S1R	(121)
Withaferin A	SLC7A11	Inhibition of Nrf2 and SLC7A11	(122)
Aspirin	SLC7A11	Inhibition of SLC7A11 transcription	(123)

↑ indicates more LPO accumulation, and ↓ indicates a decrease in GSH content.

### 4.1 Nanoparticles

HCC is difficult to detect in the early stages of clinical work due to examination methods and clinical manifestations limitations. Once diagnosed, it progresses into advanced hepatocellular carcinoma that is extremely resistant to treatment and has unsatisfactory therapeutic results. Therefore, the need for innovative therapeutic approaches is urgent, and in recent years, targeted or chemotherapeutic drug delivery approaches using nanoparticles have started to emerge. Tang et al. (124) discovered that iron-catalyzed apoptosis might be induced by the degradation of manganese-silica nanoparticles themselves (through breakage of Mn-Obond). Manganese-silica nanoparticles loaded with sorafenib were created and combined with the drug’s effects. Significant anti-tumor effects were produced when applied to HCC cells. Similar to the above, Liu et al. (125) created an iron-apoptosis-associated therapy for hepatocellular cancer using a sorafenib-loaded MIL-101 (Fe) nanomedicine that was delivered in conjunction with iRGD. The neurociliin-1 (NRP-1) receptors are largely found in tumor and vascular tissues. The iRGD peptide preferentially binds to these receptors, activating the endocytosis transport route and facilitating drug penetration into tumor tissues (125). This procedure dramatically raised lipid peroxidation and malondialdehyde levels, increased auto-induced iron apoptosis in HepG 2 cells, and lowered glutathione and glutathione peroxidase 4 (GPX4) levels. This combination (125) have several promising characteristics including drug-loading, controllable release, peroxidase activity, biocompatibility, and T2 magnetic resonance imaging ability. One of the primary palliative treatment options for advanced hepatocellular carcinoma is transcatheter arterial chemoembolization (TACE). However, TACE creates a hypoxic microenvironment in the tumor that reduces the sensitivity of chemotherapy and plays a significant role in the unpredictable treatment outcome (126). Gelatin was used as the wall material for the microspheres during the preparation of adriamycin/triiron tetraoxide composite gelatin microspheres (ADM/Fe3O4-MS), and it was confirmed (127) that it is possible to use uniformly sized gelatin microspheres coloaded with-Fe3O4 nanoparticles and adriamycin to increase the effectiveness of TACE in the treatment of HCC.

### 4.2 Phytochemicals

Active substances derived from plants are referred to as phytochemicals. Examples include polysaccharides, polyphenols, and alkaloids. Numerous studies have demonstrated the exceptional anti-tumor efficacies of numerous phytochemicals, including baicalin and curcumin, with less adverse effects when compared to other chemotherapy medicines (128). Numerous phytochemicals have been employed as ferroptosis modulators in HCC cells to far. By encouraging iron apoptosis through the increase of ER stress and the production of PEBP1/15-LO, DHA can limit the progression of HCC (129, 130). Additionally, Heteronemin and Artesunate increased ROS and promoted ferritin degradation to induce ferroptosis, respectively, to enhance the anticancer activity of sorafenib against HCC (131). An isolated substance from Lonergania japonica called lonerganine has anti-infective properties. Lonerganine was originally used to treat HCC by Jin et al. (132). Metabolomics and a mouse xenograft model both supported the findings that lobelia alkaloids reduced tumor volume and weight and prevented hepatocyte invasion and migration. By inhibiting GPX4 and GSS, solasonine increased the amounts of lipid ROS in HepG2 cells to produce these effects (132).

### 4.3 Chemotherapy

Despite the advent of novel and rising HCC therapeutic techniques during the last two decades, chemotherapy remains the anticancer treatment pioneer. It is employed as a first-line therapy method for the majority of advanced cancers (133), but drug resistance poses a significant obstacle to tumor treatment. Sorafenib blocks systemic XC-mediated cystine input, causing endoplasmic reticulum stress, GSH depletion, and iron-dependent lipid ROS buildup (114). Nrf2 and metallothionein-1G (MT-1G) are two critical variables in sorafenib-resistant HCC (87). Sorafenib causes the production of P62, which inhibits NRF2 degradation and promotes the inactivation of KEAP1, followed by the activation of heme oxygenase-1 (HO-1), quinone oxidoreductase-1, and ferritin heavy chain-1, which enhances NRF2 nuclear localization. Sorafenib stimulates NRF2 expression in HCC cells, which boosts MT-1G expression, which prevents ferroptosis by inhibiting GSH depletion, which is one of the pathways associated in sorafenib resistance (87). Inhibiting any of the aforementioned pathways might thus theoretically accelerate ferroptosis in HCC cells. An iron-sulfur protein is CDGSH iron-sulfur structural domain 2 (CISD 2). CISD 2 inhibition improved sorafenib-induced iron apoptosis in drug-resistant cells via inhibiting ferritin phagocytosis or the p62-Keap1-NRF2 pathway (95).

Metformin is commonly used to treat diabetes, and its involvement in malignancies has been studied extensively. Metformin has been shown (134) to induce iron apoptosis and increase ROS levels in hepatocellular carcinoma cells via ATF4/STAT3, thereby decreasing hepatocellular carcinoma cell resistance to sorafenib. Meanwhile, metformin used with other medications to treat malignancies has produced impressive effects. Tang et al. (135) demonstrated that metformin mixed with sorafenib promoted iron apoptosis via the p62-Keap1-Nrf2/HO1 signaling pathway, inhibiting the value-added of HCC cells.

Unfortunately, chemotherapy alone is often unsatisfactory, with intrinsic and acquired resistance to conventional chemotherapeutic agents and targeted drugs developing in a short period of time. This largely limits the efficacy of the drugs and the survival of the patients. Therefore, the use of combination therapy is a viable path. A meta-analysis by Li et al. (136) demonstrated that the efficacy of combination therapy was superior to that of monotherapy. Interventional therapy, which promotes ischemic necrosis of tumor tissues by blocking the blood-supplying arteries of the tumor, has become the optimal choice for patients with advanced unresectable hepatocellular carcinoma. Drug-eluting microspheres have been widely used in the treatment of hepatocellular carcinoma for the simultaneous effects of embolization of blood vessels supplying tumors and sustained release of chemotherapeutic agents (137). However, in a hypoxic environment, vascular endothelial growth factor and HIF-1α are activated in tumor cells, increasing tumor neovascularization and the likelihood of metastasis. Ionizing radiation from radiation therapy also induced HIF-1α accumulation, which resulted in nuclear translocation of SLC7A11 and inhibited ferroptosis in HCC (138). In this situation, combination therapy shows unique advantages, CPT (an NRF2 inhibitor) (139), anti-angiogenic drugs (140), immune drugs (PD-1 inhibitors), etc. (141) in conjunction with chemotherapeutic drugs can reduce the resistance of tumor tissues.

### 4.4 ncRNA

Noncoding RNA (ncRNA) studies have revealed that ncRNAs such as microRNAs, long noncoding RNAs (lncRNAs), and circular RNAs play important roles in the carcinogenesis and progression of HCC. These non-coding RNAs are involved in tumorigenesis and progression and affect ferroptosis (142). Two homeoboxes Through activities on the Fork head Box M1 (FoxM1) protein, A pseudogene 8 (DUXAP8), a pseudogenederived lncRNA, may promote pancreatic cancer, non-small-cell lung cancer, and HCC. LncRNA DUXAP 8 is overexpressed in hepatocellular carcinoma and is associated with a poor prognosis, possibly contributing to sorafenib resistance by suppressing ferroptosis (143). Shi and colleagues (143) explored whether DUXAP 8 reduced HCC vulnerability to sorafenib-induced ferroptosis by acting on SLC7A11. Through binding to SLC7A11, DUXAP 8 decreases membrane translocation and facilitates sorting of depalmitoylated SLC7A11 to lysosomes. Thus, suppressing DUXAP8 accelerates iron mortality in HCC, and combining it with sorafenib improves treatment efficacy in patients with advanced HCC. The above results were demonstrated in CDX models at the same time. According to a recent study (144), the lncRNA HEPFAL acts on SLC7A11 and reduces its expression, resulting in a decrease in the amount of cystine transported into the cell and, as a result, a decrease in the intracellular GSH level, which effectively inhibits the iron apoptosis function of GPX4 and, ultimately, iron apoptosis in tumor cells. In the meantime, the PI3K-AKT-mTOR signaling pathway is involved in this process. Mechanistically, it could be because PI3K activates AKT via downstream molecules, and AKT activates mTOR by phosphorylation. mTORC1 is activated by AKT and governs ferroptosis through modulating a number of molecules, including SLC7A11, GPX4, and ferroportin, primarily via NRF2. In conclusion, these should be investigated further as additional research uncover the significance of RNA in ferroptosis in HCC.

## 5 Concluding remarks and future perspectives

There is mounting evidence that ferroptosis activation or inhibition has a significant impact on a variety of illnesses and could be used to treat HCC. In recent years, an increasing variety of medicines and newly created materials have been used in experimental animals with good efficacy. In the meantime, the formation of ferroptosis resistance during treatment is a new issue. Several regulatory mechanisms in the ferroptosis process protect cells from ferroptosis inhibition. Because a single ferroptosis pathway is more likely to trigger resistance to ferroptosis, multiple pathways should be coupled to promote ferroptosis in tumor cells at the same time. Furthermore, addressing an ferroptosis factor that causes damage to normal tissues, such as kidney injury and neurotoxicity, should not be disregarded. Creating various drug delivery channels is also an option, as is improving drug delivery, drug biodistribution, and pharmacokinetics.

## Author contributions

XL: Writing – original draft, Writing – review & editing. FM: Writing – review & editing. HW: Writing – review & editing. LS: Writing – review & editing. SC: Writing – review & editing. FC: Writing – review & editing. GL: Writing – review & editing.
